# Funding model for embedded research: Impacts, challenges, and lessons learned for investigators

**DOI:** 10.1002/lrh2.70015

**Published:** 2025-04-26

**Authors:** C. Ann Vitous, Kaylee W. Burgan, John P. Donnelly, Nicholas W. Bowersox, Linda M. Kawentel

**Affiliations:** ^1^ VA QUERI Center for Evaluation and Implementation Resources (CEIR) Ann Arbor Michigan USA; ^2^ VA HSR Center for Clinical Management Research Ann Arbor Michigan USA; ^3^ Department of Learning Health Sciences University of Michigan Ann Arbor Michigan USA; ^4^ Department of Psychiatry University of Michigan Ann Arbor Michigan USA

**Keywords:** embedded research, funding models, learning health systems

## Abstract

**Introduction:**

Embedding research mechanisms within Learning Health Systems can improve healthcare performance by informing program development and expansion, supporting strategic planning, and demonstrating value. The role of the investigator is crucial in partnered funding mechanisms, and there is a growing need to better understand how investigators operate in this space. This evaluation assessed the impacts, challenges, and lessons learned by investigators funded through an embedded research program.

**Methods:**

This was an outcome evaluation within the Department of Veterans Affairs healthcare system and followed an adapted version of the Centers for Disease Control and Prevention evaluation framework. Three data sources were used to generate information: a descriptive analysis of close‐out reports, a comprehensive search of research databanks, and an online survey focused on high‐priority areas. Participants were investigators whose projects took place during the FY2017–2021 funding period. During the funding period, 25 unique investigators completed projects.

**Results:**

Investigators indicated that the embedded research program had significant, meaningful impacts on their professional development, including generating tangible products that advanced their professional goals and more subtle impacts, such as increasing knowledge and experience, increasing multidisciplinary collaboration, and creating opportunities for professional development. Investigators also reflected on challenges, including issues with the data, logistical concerns, and disruptions due to COVID‐19. Investigators identified lessons learned for future collaboration efforts, including the need for feasible and appropriate goals, as well as dedicated and trained support staff.

**Conclusions:**

The insights from this evaluation provide understanding on the impacts, challenges, and lessons learned from the experiences of investigators participating in embedded research mechanisms. The impacts illuminated explicit ways in which investigators professionally benefited from this program, as well as challenges and lessons learned that offer insights into how future programs can be tailored. These findings offer ways to improve practices in implementing LHS in other large healthcare organizations.

## 1 INTRODUCTION

There are often significant delays in translating healthcare research into practice.^1^ This is a pressing problem given that many of the challenges facing healthcare are occurring rapidly and often in an uncoordinated manner in response to market, political, social, and public health forces.^2^ The Learning Health System (LHS), defined as “a health system in which internal data and experience are systematically integrated with external evidence,” has emerged as one conceptual strategy to address these challenges.^3^, ^4^, ^5^, ^6^

One fundamental mechanism of a LHS is embedding research within the health system to improve the impact and timeliness of research and facilitate the uptake and implementation of the evidence generated.^4^ Embedded, or partnered, research is defined as the process by which research investigators and health system partners “work together, with different roles, to use research both to solve practical problems and contribute to science.”^7^, ^8^ Literature suggests that embedding research mechanisms within LHSs can improve healthcare performance by informing program development and expansion, supporting strategic planning, and demonstrating value.^4^ However, partnered research also presents challenges, such as a disconnect between healthcare investigator and operational leadership priorities.^9^ While previous literature has focused on the contributions of embedded investigators in LHSs, a lack of studies focus on the benefits of these partnerships for investigators.^10^ The role of the investigator is crucial in partnered funding mechanisms, and there is a growing need to better understand how embedded investigators operate in this space.^11^

This study presents the findings of an evaluation of the Veterans Affairs (VA) Health Systems Research (HSR) Researchers and Evaluators in Residence (REiR) program, one of several embedded research mechanisms that VA leadership developed to support its transition to a LHS.^5^ REiR projects provide funding support to investigators, which allows them to create integrated study teams with clinical personnel to generate information to inform efforts by national clinical program offices (i.e., specific organizational units or departments responsible for managing and overseeing a particular program or set of programs related to Veteran benefits, healthcare, or other services) to address areas of concern.^12^

The REiR program has a unique structure that incorporates features associated with career development awards—such as investigator funding, competitive applications, merit‐based reviews conducted by research leadership—with partnered evaluation collaborations. This includes generating questions of interest from operational leaders and fostering direct collaboration between investigators and operational personnel, with the expectation that findings will be directly applied within VA. REiR projects are expected to generate research products like manuscripts and posters, as well as operational products like summary reports and briefing slide sets. Additionally, REiR projects are expected to set the stage for additional investigator‐initiated work through both grantsmanship and collaboration with operational leaders. While this approach has similarities with embedded research programs outside of VA, there are also key differences. For instance, the Agency for Healthcare Research and Quality and the Patient‐Centered Outcomes Research Institute have funded Learning Health System Embedded Scientist Training and Research (LHS E‐STaR) centers.^6^ These centers are formal training programs which aim to train learning health scientists in embedded research methods. This is distinct from the REiR program in that it is structured as a training program and does not have a specific focus on quality improvement within a single integrated health system.

The REiR program was designed to allow program offices to benefit from investigators' expertise in research methods while also enabling investigators to better understand important policy questions and lay the groundwork for larger‐scale projects.

## 2 QUESTION OF INTEREST

The evaluation assessed impacts, challenges, and lessons learned by investigators funded through an embedded funding mechanism with a particular focus on the benefits that these investigators received from participating in partnered research projects. This knowledge can help improve practices in recruiting and retaining embedded investigators and implementing LHSs in other large healthcare organizations.

## 3 METHODS

### 3.1 Study design

This study was designed as an outcome evaluation and followed an adapted version of the Centers for Disease Control and Prevention's (CDC) Framework for Program Evaluation in Public Health.^13^ It was designated as a non‐research quality improvement project in accordance with Veterans Health Administration (VHA) Program Guide 1200.21^14^ and was exempt from IRB oversight. However, steps were taken to ensure that the safety and welfare of participant data were appropriately protected. Data were kept on a secure VA server. Findings were deidentified to protect confidentiality. Throughout the evaluation, authors examined their own subjective point of view and identified how it could impact the outcomes of the evaluation (e.g., backgrounds, training, personal experiences).

### 3.2 Participant sampling

Participants were REiR investigators whose projects took place during the FY2017–2021 funding period. During the funding period, 25 unique investigators completed REiR projects.

### 3.3 Data collection and analysis

Three data sources were used to generate information related to impacts, challenges, and lessons learned for REiR investigators: a descriptive analysis of close‐out reports, a comprehensive search of research databanks, and an online survey focused on high‐priority areas. Data from HSR was also used to provide context on investigator characteristics. Table 1 provides an overview of data sources.

**TABLE 1:** Data collection and analysis sources.

Data source	Number of investigators	Analytic process	Findings
HSR data	25	Descriptive characteristics	Investigator demographics
Close‐out forms	25	qualitative descriptive content analysis	Impact, lessons learned, challenges
Review of research databanks	25	Literature search	Impact
Brief survey	15	Descriptive analysis	Impact

#### 3.3.1 Close‐out reports

Close‐out reports included mid‐year, end‐of‐year, and extension request reports from 25 unique investigators who worked on HSR‐funded REiR projects during the period being evaluated (FY2017–2021). Close‐out reports included information on (1) the background and purpose of the REiR project, (2) work completed, including existing products, (3) direct and potential impacts of the work completed, (4) key successes and lessons learned, (5) plans for future work with program partners, (6) feedback on the project experience, and (7) feedback on the overall REiR program.

Descriptive content analysis was used to analyze the data of close‐out reports. Close‐out reports were read by one author (LK) and an initial codebook was created. Next, two additional evaluation team members (CAV, KB) read through a cross‐section of reports, adjusting the codebook as necessary. Deductive and inductive codes were created. Deductive structural codes were derived from the guiding evaluation questions. Inductive codes were also created to allow insight that was initially beyond the evaluators' purview. Definitions of each code were created and revised as necessary throughout the coding process. Two authors (CAV, KB) independently coded five close‐out reports, blinded to the other's work. Coders met to compare, review the code structure, and revise the codebook as necessary. The remaining transcripts were then divided and independently coded between the two coders. Secondary coding occurred in 25% of the reports. Differences were resolved through consensus. A descriptive matrix was used to synthesize all relevant responses.^15^

#### 3.3.2 Research databanks

Three evaluation team members (NB, CAV, KB) searched the published literature related to the REiR program within PubMed and Google Scholar for all REiR investigators during the evaluation period of interest. Search terms included investigator and topic‐specific combinations of text words, and the date range was between 2017 and current. Peer‐reviewed studies that were presumed to be connected to the REiR program were included. All potential publications for each investigator were compiled in a list. The list of publications was sent to each respective investigator, asking them to confirm and add to the list as appropriate via a survey.

#### 3.3.3 Survey

A voluntary, confidential survey was sent to all REiR investigators who were funded during the period of interest through Qualtrics, a VA‐approved web‐based survey platform. Survey items focused on areas of high priority that were not adequately covered in other data sources, such as products produced by the investigator as part of REiR participation (e.g., peer reviewed publications, reports to program partners), the extent to which different aspects of professional development were impacted by REiR participation, likelihood to recommend the REiR program to other investigators, research awards received since participating in the REiR program, and whether investigator and program partner expectations related to the program were met. The survey consisted of closed‐ended questions and one open‐ended question.

Close‐ended survey data was analyzed in STATA 18.0 MP (StataCorp) using descriptive analysis. The open‐ended item was analyzed using an iterative approach to descriptive content analysis.^16^ Responses were independently coded by two evaluation team members (CAV, KB) using the same code book that was used for the close‐out forms from REiR investigators. Coding discrepancies were resolved through consensus.

## 4 RESULTS

Among all REiR investigators in the funding period (*n* = 25), most reported their academic rank at the time of application as Assistant Professor (52%), followed by Associate Professor (24%) or other academic ranking (24%). All REiR investigators completed close out reports, and 15 participated in the survey (63% response rate).

Data from close‐out reports, research databanks, and surveys illuminated impacts, challenges, and lessons learned from investigators participating in the REiR program.

### 4.1 Impact of REiR participation on investigators

In close‐out reports, REiR investigators discussed several impacts to their professional development, specifically, building connections, increasing understanding of data sources, and advancing scholarship and strengthening research skill set.

One key impact of the program reported by investigators was the opportunity for building connections across disciplines, offices, and community partners (i.e., organizations and individuals that work with VA facilities and healthcare providers to improve communication, coordination of care, and access to resources for Veterans). They reported that their REiR project allowed them to expand their clinical, research, and community networks, and allowed them to connect with offices and investigators they would not have had the chance to otherwise. For many, the opportunity to develop such relationships was one of the primary strengths of participation.

The REiR position gave me the opportunity to connect with VA researchers across the country, with whom I would not have worked with without the REiR position. I believe these relationships will lead to future collaborations to improve policies around women Veterans' employment and increasing access to employment opportunities. (ID10)

Another key impact of the program was the opportunity for an increased understanding of data sources. This was reported as not only gaining training on additional data repositories but also how that data impacted decision making.

It has given me a much better sense of how data can inform decision making and reinforced the importance of measurement and shortcomings of electronic health record data. It gave me the opportunity to better understand how VHA leaders think and how my work can be most useful for decision making. (ID12)

Correspondingly, several investigators indicated that their REiR projects helped advance scholarship, either by setting the stage for additional work which extended the work related to their REiR project or by helping to identify areas for future inquiry more generally. Notably, a handful of investigators reported that their participation in the REiR program directly led to the development and/or submission of merit‐based grant applications and fellowships to extend their REiR projects or work alongside their collaborators on similar projects. Additionally, participants noted that participation allowed them to strengthen their quantitative and qualitative skillsets.

In addition to what was described in close‐out reports, REiR investigators were asked about how the REiR program impacted different aspects of their professional development in the survey. For 7 out of 10 areas that were assessed, more than half of investigators reported that participation in the REiR program impacted that area “quite a bit” or “a great deal.” These professional development areas included opportunities to contribute to VA's mission (93%), building connections with program offices (80%), increasing understanding of data sources (66%), increasing understanding of VA priorities (66%), providing opportunities to advance scholarship (59%), and strengthening research skillsets (59%). Investigators were less likely to endorse that REiR participation contributed “quite a bit” or “a great deal” to opportunities to directly contribute to clinical practice (33%), providing pilot data to support scale‐up (33%), and direct funding opportunities with program offices (26%). Table 2 provides an overview of reported impacts on areas of professional development.

**TABLE 2:** Reported impacts on areas of professional development (survey).

	Other impact^a^		“Quite a bit” or “A great deal” impact	
Impact	*N*	%	*N*	%
Allowed you to contribute to VA's mission	1	6.7%	14	93.3%
Helped you build connections with VA operational offices	3	20%	12	80%
Furthered your professional career goals	4	26.7%	11	73.3%
Increased your understanding of VA data sources	5	33.3%	10	66.7%
Increased your understanding of VA national priorities	5	33.3%	10	66.7%
Provided you an opportunity to advance your scholarship	6	40.0%	9	60.0%
Strengthened your research skillsets	6	40.0%	9	60.0%
Provided pilot data to support the scale‐up of an early‐stage study	10	66.7%	5	33.3%
Allowed you to make direct contributions to clinical practice	10	66.7%	5	33.3%
Led to direct funding opportunities with VA operational offices	11	73.3%	4	26.7%

^a^ Other impact responses included “Not at all,” “A little,” or “A moderate amount.”

Investigators also reported on specific products that resulted from REiR efforts. They indicated that REiR projects contributed to manuscripts (*n* = 17), conference presentations (*n* = 22), reports (*n* = 9), and briefings (*n* = 12). In addition to the products verified by investigators, the review of research databanks yielded an additional three manuscripts from investigators who did not participate in the survey. Frequency counts of all products can be found in Table 3.

**TABLE 3:** Frequency counts of products, FY2017–FY2021.

Deliverable	Unique investigators	Total
Published manuscripts	13	17
Academic conference presentations	7	22
Internal summary report to VA operational leadership	6	9
Internal briefings to VA partners and leadership	7	12
		60

REiR investigators were asked about the types of funding awards they have received as a principal investigator (PI) or co‐principal investigator (Co‐PI) since the completion of their REiR. Twelve unique investigators reported receiving funding, with some reporting having received multiple additional sources of funding (Table 4). Notably, this reporting focused on funding more generally and not solely on those funding awards that directly resulted from REiR project work.

**TABLE 4:** Type of funding after REiR^a^ ^,^ ^b^.

Type of funding	After REiR (N)
Career development award	2
Merit review award	2
Special office of research and development funding solicitation	2
Health systems research investigator initiated research (investigator initiated research) project	5
Other	8
None	3
Total	22

^a^ Only includes confirmed products from survey respondents (*n* = 15).

^b^ Some investigators received multiple forms of funding; totals are more than 100%.

Finally, in terms of impacts, 87% (*n* = 13) of investigators who participated in the survey provided open‐ended responses related to the ways that their REiR projects contributed to other important outcomes. Several were able to speak directly to how their work was being used by program offices, while others reemphasized the professional development opportunities associated with participation in REiR, such as directly leading to additional funding or supporting further relationship development with partners, investigators, and community networks, reinforcing findings from close‐out reporting documents.

The project conducted during the REiR program had a great deal of visibility and [redacted] was able to state that novel methods will be added to their analytic tools for future efforts. Moreover, the work completed during this residency allowed for VA to have more confidence in the safety of the COVID‐19 vaccine and we were able to present and discuss this with not only leadership and other agencies but with the providers in the field having direct contact with patients … These analyses [target trial emulation to study vaccine safety] are now part of the formal process of conducting full evaluations when warranted on specific drug safety questions. (ID23)

I was already highly connected with my operations partners when I was awarded the REiR project. We continue to work together in a highly partnered way and all of my research, including the results of the REiR, are used by their office. (ID20)

### 4.2 Challenges associated with participation in the project

Despite the positive impacts to professional development, investigators described various challenges associated with their participation in the REiR program in close‐out reports. Specific challenges included issues with the data, logistical and administrative concerns, and disruptions due to COVID‐19.

Some investigators reported issues with data. Sometimes, the challenge was a delay in accessing the necessary data. In other instances, there was a mismatch between what investigators expected to be able to learn from the data and what the data offered. This was often due to data that was missing, incomplete, or not structured in standardized ways. As one investigator asserted, “*The data was in the relatively early stages of being cleaned and organized, therefore we experienced some limitations with regard to what we could investigate*.” (ID2) Finally, in one instance, there was a lack of storage space for the data. Although investigators found workarounds for most of these challenges, it had the impact of causing delays and, in some cases, causing the collaborators to need to modify the questions being asked.

Logistical and administrative challenges were also identified as a concern. Logistical challenges included the inability to attend Health Systems Research/Quality Enhancement Research Initiative (HSR QUERI) meetings (which were expected to provide opportunities for enhanced, direct collaboration on REiR project work) due to scheduling conflicts, canceled meetings, difficulty getting on calendars and missing scheduled trips due to unforeseen circumstances (e.g., health, COVID‐19). As one investigator described, “*A lesson learned is to plan ahead for meeting/collaboration times. It is difficult to get on calendars due to busy schedules and competing job duties*.” (ID4) Other challenges included insufficient protected time, evaluation activities being too dependent on factors outside investigators' control and hiring delays for support staff. As one investigator described, “*I did not realize that my ability to complete the planned evaluation activities would be dependent on program related issues that were outside of my control*.” (ID6) Although many investigators were able to overcome or work through these challenges, they often resulted in project delays or confusions.

Notably, based on the timing of these REiR projects, many were impacted by COVID‐19. For some, it interrupted the ability to organize, leading to data collection delays, shifts in work processes, and competing demands (e.g., effort being reallocated). For others, it led to the scaling back of intervention and evaluation activities regarding the goals and methods employed (e.g., semi‐structured interview versus ethnography).

The primary challenges over the past 3 months are due to the COVID‐19 environment, which has led to a scaling back of the intervention and evaluation, and changes in the way curriculum is being delivered. (ID6)

### 4.3 Lessons learned from participating in REiR

Investigators identified lessons learned for future collaboration efforts, including the need for feasible and appropriate goals and dedicated and trained support staff.

Feasible and appropriate goals based on the REiR program were discussed as a need both at the onset and throughout the project, as articulated by one investigator, “*We should have set expectations and meeting times earlier in the project*.” (ID4) Investigators highlighted the importance of collaboratively defining project goals, ensuring that they were focused and feasible based on available time, available support staff, level of expertise, appropriately matched with the timeline of the program being evaluated, and met a significant need of the program offices. For example, one investigator asserted, “*Some of the deliverables were relatively ambitious for the one‐year timeline, particularly since we did have some initial delay gaining data access*.” (ID2) It was recommended that these goals be regularly revisited during the project, accounting for unexpected challenges such as a change in leadership or program needs. Similar conversations were recommended to be had relating to expected products (e.g., academic, and non‐academic products).

In addition, investigators asserted a need for dedicated and trained support staff. For some, this need overlapped with other logistical and administrative challenges, such as insufficient protected time, hiring delays, or inconsistent availability of support staff. For others, this overlapped with the need to have feasible and appropriate goals aligned with the data or methodological approach proposed to address the evaluation questions.

The biggest lesson was the necessity to have research skills, vocational rehabilitation expertise (formed by past and current research in this field), and data and CDW [Corporate Data Warehouse] expertise. Without significant data expertise, many of the [redacted] objectives would not have been possible. (ID1)

In some cases, this resulted in investigators hiring additional support staff and, in some instances, securing additional funding.

Researchers may not have all the skills to do the work. For example, I don't have strong enough programming skills to conduct all the statistical analyses, so I brought in an analyst to assist, but that was not originally how the program was designed. (ID12)

In other instances, investigators needed to modify or scale back the expected work. Investigators recommended that available staffing should be communicated at the onset of the project and discussed regularly to address any additional staffing needs to ensure timely project completion.

## 5 DISCUSSION

The investigator is essential in supporting the LHS. Given VA's role as the largest healthcare system in the United States, the results of this evaluation allow for insight into the experiences of embedded partnerships for investigators. This evaluation found that the REiR program had significant, meaningful impacts on their professional development, including generating tangible products that advanced their professional goals and more subtle impacts, such as increasing knowledge and experience, increasing multidisciplinary collaboration, and creating opportunities for professional development. Investigators also reflected on challenges, including issues with the data, logistical and administrative concerns, and disruptions due to COVID‐19. Investigators identified lessons learned for future collaboration efforts, including the need for feasible and appropriate goals, as well as dedicated and trained support staff.

Investigators are pivotal in advancing innovative scholarship in health services research. Figuring out how to attract and retain investigators is imperative for the future success of LHSs. While professional development often relies on investigators obtaining funding, external funding sources are often highly competitive, limited in funding, and lack collaborative approaches to research.^17^, ^18^ Moreover, external funding tends to focus on hypothesis‐driven scientific inquiry without adequately supporting the infrastructure needed for ongoing administration and data management to optimize LHS functions.^19^

These challenges pose a threat to the career development of investigators in health systems research. Embedded funding mechanisms can offer a viable alternative to traditional funding sources for investigators by offering a stable funding mechanism for high‐impact research. This approach helps strengthen research capacity, offers the ability to co‐produce research, and helps overcome persistent barriers to translating research into practice.^19^, ^20^, ^21^ Additionally, during the evaluated funding period, an investment of approximately $1.06 million resulted in an estimated 32 newly funded projects, demonstrating that a relatively small investment can lead to significant positive impacts on funding outcomes.

Despite the potential advantages, investigators face challenges. Previous research has indicated issues such as shifting priorities based on leadership changes, the tension between the need for academic products and more pragmatic products of program offices, power dynamics, misaligned timelines, and adjustments in funding priorities.^8^, ^22^ While many of these challenges were also identified in this evaluation – such as misaligned timelines and realignment in funding priorities—this evaluation provides new insights. It highlights the difficulties of mismatched skill sets and lack of control over accessing previously generated data. Furthermore, this evaluation shows that investigators can successfully produce academic products, like peer‐reviewed publications, conference presentations, and additional research funding, in addition to more pragmatic products, such as reports for program partners.

Important lessons learned can be used to offer recommendations to embedded funding mechanisms going forward. Previous studies highlight the benefits of a dedicated funding stream, the development of a research pipeline that includes short and long‐term projects, and the need for investigators to be flexible as priorities shift over time.^8^ This evaluation complements many of these lessons, particularly on the need to be flexible given the often rapidly changing priorities of healthcare systems. This evaluation also builds on these findings by demonstrating investigators' expressed desire to have feasible and appropriate goals based on the timeline, available data, and availability of resources. This suggests the need for additional administrative oversight at the onset and throughout the funding duration.

This evaluation had limitations. The lack of standardization of demographic data collected, stored, and monitored for investigators resulted in missing and incomplete data across multiple domains. Further, disruptions during the evaluation period of interest (e.g., COVID‐19 and transition of the program's management) contributed to inconsistency in data collected from investigators. Additionally, the data presented are investigators' own reports, which naturally reflect their personal points of view. Finally, although beyond the scope of this evaluation, the evaluation did not include an analysis of the dollar amount spent per measurable impact. Despite these limitations, this evaluation represents the systematic synthesis of information generated from multiple data sources to ensure an accurate representation of investigator experiences with an embedded program.

This study highlights the contributions investigators make within the systems they support. It aligns with existing research by identifying several challenges faced by these investigators, including shifting priorities due to leadership changes, power dynamics, misaligned timelines, and varying funding priorities.^8^, ^22^ However, this study reveals differences that challenge the assumption of a conflict between academic outputs and practical deliverables. Additionally, the findings indicate that investigators are eager for attainable and relevant goals that fit their timelines, available data, and resources, underscoring the need for enhanced administrative oversight throughout the funding process. These findings offer ways to improve practices in implementing LHSs in other large healthcare organizations.

## CONFLICT OF INTEREST STATEMENT

John P. Donnelly receives personal fees from the American College of Emergency Physicians as a methodology/statistics editor for *Annals of Emergency Medicine*.

## References

[1] Morris ZS , Wooding S , Grant J . The answer is 17 years, what is the question: understanding time lags in translational research. J R Soc Med. 2011;104(12):510‐520. doi:10.1258/jrsm.2011.110180 22179294 PMC3241518

[2] Health Services Research: Opportunities for an Expanding Field of Inquiry—An Interim Statement. National Academies Press; 1994:9242. doi:10.17226/9242 25101441

[3] Kilbourne AM , Goodrich DE , Miake‐Lye I , Braganza MZ , Bowersox NW . Quality enhancement research initiative implementation roadmap. Med Care. 2019;57(10 Suppl 3):S286‐S293. doi:10.1097/MLR.0000000000001144 31517801 PMC6750196

[4] Vashi AA , Orvek EA , Tuepker A , et al. The Veterans Health Administration (VHA) innovators network: evaluation design, methods and lessons learned through an embedded research approach. Healthcare. 2021;8:100477. doi:10.1016/j.hjdsi.2020.100477 34175094 PMC8244154

[5] Kilbourne AM , Schmidt J , Edmunds M , Vega R , Bowersox N , Atkins D . How the VA is training the next‐generation workforce for learning health systems. Learn Health Syst. 2022;6(4):e10333. doi:10.1002/lrh2.10333 36263263 PMC9576233

[6] About Learning Health Systems. Accessed October 31, 2024. https://www.ahrq.gov/learning-health-systems/about.html

[7] Ovretveit J , Hempel S , Magnabosco JL , Mittman BS , Rubenstein LV , Ganz DA . Guidance for research‐practice partnerships (R‐PPs) and collaborative research. J Health Organ Manag. 2014;28(1):115‐126. doi:10.1108/JHOM-08-2013-0164 24783669

[8] Gould MK , Sharp AL , Nguyen HQ , et al. Embedded research in the learning healthcare system: ongoing challenges and recommendations for researchers, clinicians, and health system leaders. J Gen Intern Med. 2020;35(12):3675‐3680. doi:10.1007/s11606-020-05865-4 32472492 PMC7728937

[9] Bowersox NW , Williams V , Kawentel L , Kilbourne AM . Sustaining effective research/operational collaborations: lessons learned from a National Partnered Evaluation Initiative. Healthcare (Amst). 2021;9(4):100588. doi:10.1016/j.hjdsi.2021.100588 34823223

[10] Damschroder LJ , Knighton AJ , Griese E , et al. Recommendations for strengthening the role of embedded researchers to accelerate implementation in health systems: findings from a state‐of‐the‐art (SOTA) conference workgroup. Healthcare. 2021;8:100455. doi:10.1016/j.hjdsi.2020.100455 34175093 PMC8243415

[11] Vindrola‐Padros C , Pape T , Utley M , Fulop NJ . The role of embedded research in quality improvement: a narrative review. BMJ Qual Saf. 2017;26(1):70‐80. doi:10.1136/bmjqs-2015-004877 PMC5256405 27129492

[12] Researchers‐in‐Residence‐RFA.pdf. Accessed October 31, 2024. https://www.hsrd.research.va.gov/funding/Researchers‐in‐Residence‐RFA.pdf

[13] Braganza M , Kilbourne A . Quality Enhancement Research Initiative (QUERI) Evaluation Guide. 10.1007/s11606-020-06143-z PMC7878630 32875498

[14] Department of Veterans Affairs Office of Research & Development Program Guide: 1200.21 VHA Operations Activities That May Constitute Research. Accessed September 1, 2023. https://www.research.va.gov/resources/policies/ProgramGuide-1200-21-VHA-Operations-Activities.pdf

[15] Averill JB . Matrix analysis as a complementary analytic strategy in qualitative inquiry. Qual Health Res. 2002;12(6):855‐866. doi:10.1177/104973230201200611 12109729

[16] Sandelowski M . What's in a name? Qualitative description revisited. Res Nurs Health. 2010;33(1):77‐84. doi:10.1002/nur.20362 20014004

[17] Sung NS , Crowley WF , Genel M , et al. Central challenges facing the National Clinical Research Enterprise. JAMA. 2003;289(10):1278‐1287. doi:10.1001/jama.289.10.1278 12633190

[18] Adams MCB , Bicket MC , Murphy JD , Wu CL , Hurley RW . Opportunities and challenges for junior investigators conducting pain clinical trials. Pain Rep. 2019;4(3):e639. doi:10.1097/PR9.0000000000000639 31372584 PMC6675408

[19] Kilbourne AM , Borsky AE , O'Brien RW , Braganza MZ , Garrido MM . The foundational science of learning health systems. Health Serv Res. 2024;59(6):1475‐6773.14374. doi:10.1111/1475-6773.14374 PMC11622276 39165034

[20] Akintola A , Newbury‐Birch D , Kilinc S . Bridging the gap between research evidence and its implementation in public health practice: case studies of embedded research model. BMC Public Health. 2024;24(1):1299. doi:10.1186/s12889-024-18727-z 38741039 PMC11092060

[21] Haun JN , Tomoyasu N , Paolicelli C , Melillo C , Atkins D . High‐risk, high‐impact health services research: developing an innovation initiative. J Veterans Stud. 2020;6(1):171. doi:10.21061/jvs.v6i1.169

[22] McGinity R , Salokangas M . Introduction: ‘embedded research’ as an approach into academia for emerging researchers. Manag Educ. 2014;28(1):3‐5. doi:10.1177/0892020613508863

